# Spectrum of Inpatient Pediatric Dermatology Cases at a Tertiary Health Care Centre in South India

**DOI:** 10.7759/cureus.65062

**Published:** 2024-07-21

**Authors:** Sanmitra Aiholli, Arun Inamadar, Keshavmurthy A Adya, Devavrat Gore

**Affiliations:** 1 Department of Dermatology Venereology and Leprosy, Shri B. M. Patil Medical College Hospital and Research Centre, Bijapur Lingayat District Education (BLDE) (Deemed to be University), Vijayapura, IND

**Keywords:** hereditary disorders, childhood psoriasis, inpatient dermatology, pediatric dermatology emergency, pediatric dermatology

## Abstract

Background

Dermatologists and pediatricians commonly encounter pediatric dermatology cases in their clinical practice, and the number has risen for the past decade. While numerous studies have addressed adult inpatient dermatology cases, there is a lack of data on the same for the pediatric population.

Aim

The study aimed to investigate the spectrum and outcomes of inpatient pediatric dermatology cases at a tertiary health care center.

Methods

This was a hospital-based, cross-sectional study that included children under the age of 16 years with primary skin disorders who were admitted to the pediatric dermatology unit. Patients were categorized into six groups based on their provisional diagnosis for better analysis.

Results

A total of 105 children were admitted, with a male-to-female ratio of 1.2:1. The average age of admitted children was 5.8 years, with the majority belonging to the school-going age group, accounting for 44% of the patients.

Conclusion

Inflammatory skin conditions like childhood psoriasis and erythroderma were the most common group of disorders presented to us, followed by hereditary conditions like keratinization disorders and mechanobullous disorders. Pediatric dermatology emergencies (PDEs) require an inter-professional approach for timely intervention and management.

## Introduction

Dermatology has primarily been an outpatient specialty with varying morbidity and low mortality. However, dermatological emergencies need urgent inpatient care and inter-professional management [1]. A dermatological emergency is defined as a skin disorder that needs an early diagnosis, hospitalization, and careful monitoring to reduce morbidity and mortality [2]. Many hospitals lack infrastructure for inpatient care of severe skin diseases, and the number of skin disorders that require inpatient care is on the rise, especially in children [3].

Skin diseases account for 30% of all outpatient consultations with a pediatrician, while children account for 30% of outpatient dermatologist visits [4]. About 4-6% of all pediatric emergency cases account for pediatric dermatologic emergencies (PDEs), and only 30% of these represent true emergencies [5,6]. Many of the emergency physicians and pediatric residents have a limited understanding of cases presenting to PDEs and show difficulty in diagnosing and managing them effectively. The relative diagnostic agreement between dermatologists and pediatricians for skin disorders in children has been poor, at 8-66% [7].

Pediatric dermatology is a growing sub-specialty, and there is a lack of literature regarding the clinical profile of inpatient pediatric patients. Also, certain non-emergency conditions require hospital admission for thorough multiprofessional care and evaluation. This study aimed to evaluate the spectrum of inpatient pediatric dermatology cases and their outcomes in a tertiary care center.

## Materials and methods

It was a hospital-based, cross-sectional study conducted in a tertiary care hospital for two years. All children (<16 years of age) with primary skin disorders admitted to the Department of Dermatology were included in the study after obtaining written informed consent from their parents. All patients above 16 years of age and those who did not consent were excluded from the study. Detailed demographic data, history, spectrum, and duration of skin disease, associated systemic complications, duration of hospital stay, and clinical outcome of the disease were recorded. Patients were classified into six groups based on their diagnosis. The criteria for systemic inflammatory response syndrome (SIRS) proposed by Goldstein et al. were evaluated to determine the severity of systemic inflammation [8]. The data were entered in a Microsoft Excel sheet, and statistical analysis was performed using Statistical Package for the Social Sciences (SPSS) Version 20 (IBM SPSS Statistics for Windows, IBM Corp., Armonk, NY), and results are represented as frequency, percentages, and charts. Institutional ethical committee approval was obtained.

## Results

Demographics

**Figure 1:**
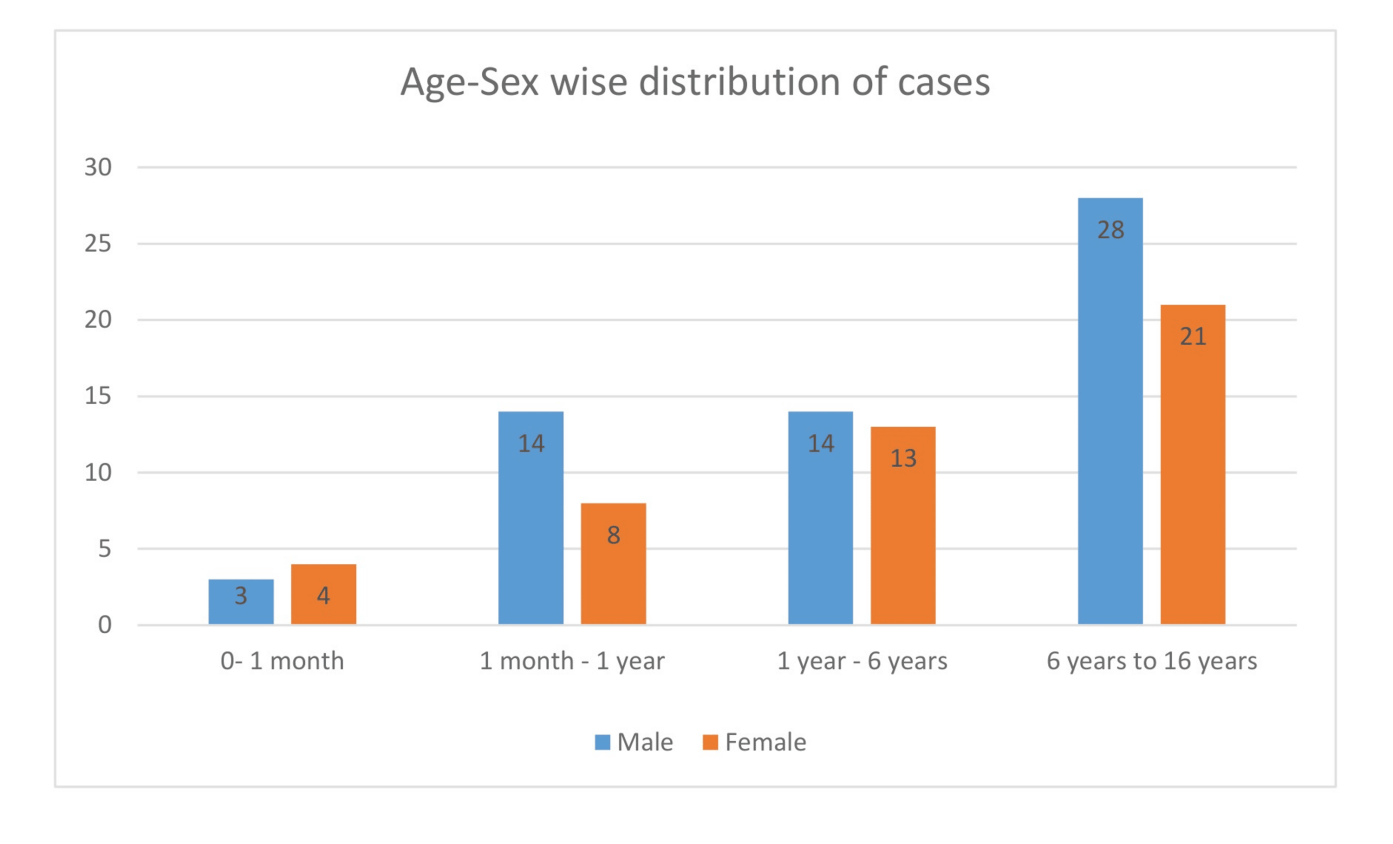
Age-sex-wise distribution of cases

A total of 105 children were included in the study. There were 57 males and 48 females, and the male-to-female ratio was 1.2:1. The age of patients ranged from one day to 16 years, and the average age of children admitted was 5.8 years. A total of 49 children admitted were in the age group of 6-16 years (47%), with 28 males and 21 females (Figure 1).

Clinical diagnosis

Spectrum of Skin Disorders

As per the diagnosis, patients were classified into six subsets of categories like cutaneous infections, inflammatory disorders, hereditary disorders, nutritional disorders, drug reactions, and other miscellaneous conditions (Table 1).

**Table 1:** The spectrum of inpatient pediatric dermatology cases *SJS/TEN, Stevens-Johnson syndrome/toxic epidermal necrolysis

Disease category (n)	Diagnosis (n)
Cutaneous infections (5)	Neonatal varicella (1)
	Purpura fulminans (rickettsial infection) (1)
	Histoid leprosy (1)
	Ecthyma gangrenosum (1)
	Impetigo with id eruption (1)
Inflammatory disorders (56)	Psoriasis (16)
	Urticaria/angioedema (10)
	Vasculitis (10)
	Erythroderma (9)
	Dermatitis (5)
	Erythema multiforme major (herpes labialis) (2)
	Lichen planus (1)
	Acute generalized exanthematous pustulosis (1)
	Sweet syndrome (1)
	Panniculitis (1)
Hereditary disorders (25)	Keratinization disorders (14)
	Mechanobullous disorders (8)
	Genetic disorders (3)
Drug reactions (2)	SJS/TEN* (1)
	Drug reaction with systemic symptoms and eosinophilia (DRESS) (1)
Nutritional disorders (4)	Acrodermatitis enteropathica (2)
	Biotin deficiency (1)
	Hartnup’s disease (1)
Others (13)	Infantile hemangioma (9)
	Alopecia totalis (1)
	Neonatal pemphigus vulgaris (1)
	Pemphigus foliaceus (1)
	Neonatal lupus erythematosus (1)

Out of 56 children (53.4%) admitted with inflammatory skin disorders, 21 cases were of childhood psoriasis. The chronic plaque type of psoriasis was the most common subtype, with six cases, followed by five children with erythrodermic and sebopsoriasis subtypes. Children with erythroderma presented with fever and raised total counts, and all patients satisfied the SIRS criteria of systemic inflammation. The average hospitalization for psoriasis cases was 7.5 days (range: 5-10 days). Urticaria with or without angioedema and cutaneous small vessel vasculitis (CSVV) were the second most common conditions, with 10 cases each (9.5%). Most children with urticaria were idiopathic, except one patient had renal calculus, and two had infectious etiology. Total WBC counts were raised in three patients, and the average duration of hospitalization was 3.9 days. Out of 10 cases of small vessel vasculitis, eight of them were in the form of Henoch-Schönlein purpura (IgA vasculitis). All children presented with a triad of palpable, non-blanchable purpuric rash over the lower limbs, abdominal pain, and hematuria. Six patients showed abnormal urine microscopy and elevated C-reactive protein (CRP) and were referred to the pediatrics department. The average duration of hospitalization was 6.7 days. A couple of cases of erythema multiforme major (EMM) secondary to herpes labialis were admitted with fever, atypical target lesions, and mucosal ulcerations over the oral cavity and were managed with a short course of intravenous systemic corticosteroids and antivirals. One patient had a recurrence and was readmitted.

Most of the genetic diseases were keratinization disorders. There were five cases of lamellar ichthyosis and three cases of both congenital bullous ichthyosiform erythroderma (CBIE) and non-bullous congenital ichthyosiform erythroderma (NBIE). A skin biopsy was collected from all cases for histopathology and genetic studies.

There were eight cases of mechanobullous disorders (12%) and three cases of other genetic disorders. A skin biopsy for histopathology and direct immunofluorescence (DIF) was obtained for all cases of mechanobullous disorders to confirm the diagnosis. The average duration of hospital stays for hereditary conditions was five days.

Five infectious conditions (4.8%) presented with high-grade fever and elevated total leukocyte counts. Weil-Felix test showed that a child with purpura fulminans (PF) had an infection caused by rickettsiae because the child had the OX-19 antigen. Another neonate presented with multiple blisters and a positive history of varicella infection in the mother before delivery, which confirmed neonatal varicella. A young adolescent came with multiple waxy, shiny nodules over the trunk and loss of sensation in the extremities. Histopathological examination confirmed histoid Hansen’s, and he was started on multidrug therapy.

Three nutritional dermatoses included two cases of acrodermatitis enteropathica (AE) and a case of biotin deficiency. Others, or miscellaneous conditions, were mainly infantile hemangioma (IH)cases.

Complications and referrals

Out of the total, 45 patients (43%) experienced systemic complications, with 14 patients (13%) developing septicemia and receiving collaborative care from the pediatrics department. A total of 35 (33%) patients were referred to the pediatrics department and one (2.5%) to the urology department for further evaluation (Table 2).

**Table 2:** Number of admissions, duration of hospital stay, and referral rates of different diagnostic categories

Clinical diagnosis (category of disease)	Number (%) of patients affected (n = 105)	Average duration of hospital stay (days)	Number (%) of patients referred (n = 45)
Inflammatory disorders	56 (53.4%)	6 (3-10)	24 (53.3%)
Cutaneous infections	5 (4.6%)	7.6 (6-10)	3 (6.7%)
Hereditary disorders	25 (24%)	4.5 (1-10)	14 (31.1%)
Nutritional disorders	4 (3.8%)	16 (6-34)	1 (2.2%)
Drug reactions	2 (1.9%)	8 (6-10)	0
Others	13 (12.3%)	4.5 (6-8)	3 (6.7%)

In all cases, SIRS was assessed upon admission. A total of 38 patients (36%) satisfied the criteria of SIRS. The average duration of hospital stay in children with SIRS and children without SIRS was 7.59 days (range: 4-34 days) and 6.26 days (range: 3-10), respectively.

Outcome

Most patients demonstrated significant improvement at the time of discharge. Parents of hereditary disorders, such as mechanobullous and keratinization disorders, were counseled about the recurrent nature of their condition and were asked to follow up regularly with the outpatient department. Five patients (5%) were transferred to the intensive care unit (ICU), and two patients (2%) left the hospital against medical advice.

Duration of stay in the hospital

The average duration of hospital stays ranged from one to 34 days, with a mean duration of 6.8 days.

## Discussion

A total of 105 pediatric dermatology cases were admitted in two years (2021-2023). In our study, boys accounted for 56% of the cases, while girls accounted for 44%, resulting in a male-to-female ratio of 1.2:1, closely resembling the ratio reported in the study by Mathias et al. [9]. The average age of inpatient children was 7.2 years, with school-going children being the most common age group among the admitted patients. Shah et al. reported 7.6 years as the average age of presentation, and Sarkar et al. recorded the highest number of children (37.9%) in the school-going age group [10,11]. The prevalence of PDEs was higher in other studies, as we did not consider skin lesions secondary to trauma, burns, or viral exanthema in our study. Moreover, there is a high probability of some cases being missed or going unnoticed, as many are initially presented to emergency physicians and pediatricians.

The prevalence of childhood psoriasis in India ranges from 0.5 to 2%. Plaque psoriasis is characterized by well-defined, erythematous to hyperpigmented plaques with silvery-white scales, and it affects 34-73% of children [12].

Erythrodermic psoriasis is a rare and severe type that accounts for 1-2% of psoriasis patients [13]. In our study, nine cases presented with fever and erythematous lesions involving more than 90% of the body surface area. The average age of patients was nine months (range: two months to two years). Sebopsoriasis was diagnosed as an underlying disorder in four out of nine patients, while plaque-type psoriasis was present in five cases. All children were immediately started on a treatment regimen comprising 5 mg/kg of oral cyclosporine, intravenous antibiotics, fluid support, and topical emollients. Most children showed improvement in erythema, scaling, and irritability within seven days. Oral cyclosporine was slowly tapered over two to three months in all cases with monthly creatinine monitoring. However, two cases were re-admitted after four months for exacerbations upon discontinuing cyclosporine. Cyclosporine therapy is useful in severe forms of psoriasis-like pustular and erythrodermic. It is also recommended to be used as a short-term crisis management drug or in resistant cases of psoriasis [14].

Sebopsoriasis is an overlap condition and has features of both seborrhoeic dermatitis and psoriasis. It can be described as a psoriatic rash with a seborrhoeic distribution, affecting young children and adolescents. There is a lack of data concerning sebopsoriasis and its outcome in the literature, and it has the potential to evolve into erythrodermic psoriasis if left untreated [15]. Oral cyclosporine (5 mg/kg) was added in cases of pustular psoriasis and sebopsoriasis to control disease activity and then tapered slowly. Weekly subcutaneous injections of methotrexate (0.3 mg/kg) were used for induction and maintenance therapy in chronic plaque psoriasis.

Acute urticaria and angioedema were the second most common disorders in our study, along with cutaneous vasculitis (10), and most cases were idiopathic. Sathishkumar et al. reported that urticaria, secondary to infections, was the most common inflammatory disorder in their study and other similar studies [16,17]. Sarkar et al. reported 12 cases of urticaria/angioedema in their study, and Auvin et al. observed 59 (15%) cases of urticaria [6,11]. Though urticaria is a benign condition, angioedema of the tongue, pharynx, and larynx can be life-threatening; hence, in-patient care is necessary to monitor and rule out infectious causes.

The prevalence of cutaneous vasculitis among children varies from 2.3 to 9%. Among 10 children admitted with CSVV in our study, eight of them presented in the form of IgA vasculitis with palpable purpura, pain in the abdomen, and microscopic hematuria. The patients improved clinically after starting systemic corticosteroids and symptomatic treatment. A couple of cases of EMM in a 15-year-old and an 11-year-old boy were seen secondary to recurrent herpes labialis and presented with atypical target lesions over extremities and oral ulcerations. Lesions subsided once oral antivirals, systemic steroids, and fluids were administered.

Hereditary conditions were the second most common group of disorders, followed by inflammatory disorders. The keratinization disorders encountered in our study include mainly lamellar ichthyosis, epidermolytic hyperkeratosis, and non-bullous ichthyosiform erythroderma. It is crucial to counsel parents on the significance of barrier repair and the necessity of frequent use of urea-based emollients, particularly during winter, for children with keratinization disorders.

Patients with epidermolytic hyperkeratosis typically present with generalized erythroderma and skin fragility within the first year of life. They require intensive, multidisciplinary care to manage dehydration, electrolyte imbalance, and skin infections like other cases of erythroderma [18].

Mechanobullous diseases are hereditary disorders characterized by multiple blisters and erosions over trauma-prone areas within a few days of birth. The mainstay of treatment remains barrier dressing with antibiotics to prevent cutaneous infections and avoid trauma. Two cases of recessive dystrophic epidermolysis bullosa (EB) presented with multiple erosions and scarring over extremities and trunks, which were confirmed by antigen mapping. These patients were treated with 25 mg of losartan tablets, which are crushed and mixed with 5 mL of cyproheptadine syrup and given at a dose of 1 mg/kg orally once a day to hasten wound healing and reduce skin scarring [19]. The new blisters gradually ceased to appear, and existing erosions started to heal within two weeks of initiating treatment. The child was followed up monthly for a total of seven months while tapering the dose. Shah et al. reported two cases of EBD and one case of EBS, while Sarkar et al. observed three cases of EB [11].

In other similar studies, infections were one of the most common PDEs recorded. Bacterial infections were more common in India and Argentina, while viral infections were most common in Western countries [20]. Children with infections characteristically present with fever and other prodromal symptoms before any cutaneous manifestations and present to pediatricians, followed by a dermatologist. Other infections, such as viral exanthema and staphylococcal scalded skin syndrome (SSSS), were not included as they were inpatients in the pediatrics department.

Out of five patients admitted for cutaneous infections, three of them developed septicemia and were jointly managed by pediatricians. A case of neonatal varicella with septicemia improved after intravenous acyclovir and antibiotic administration under intensive care. PF is a rare syndrome of intravascular thrombosis and infarction of the skin. It is commonly described with meningococcemia, staphylococcal sepsis, and spotted fever [21]. We successfully managed a case of PF secondary to rickettsial infection with intravenous doxycycline for a two-week duration. A case of extensive ecthyma gangrenosum over the trunk with fever in a neonate improved after antibiotic coverage and topical therapy. In all cases, multidisciplinary care was provided for better treatment efficacy [22].

AE is an autosomal recessive genetic disorder due to a mutation in the gene that encodes for a protein that binds with zinc in the gut. It is characterized by a clinical triad of periorificial dermatitis, alopecia, and diarrhea [23]. A 12-year-old boy presented with symmetrical annular erythematous, eczematous inflammatory plaques with discharge and crusting over the face, neck, trunk, and bilateral extremities. Further examination revealed multiple pustules along the eczematous plaques, alopecia totalis, diarrhea, and hyperpigmented patches from lesions from previous episodes. The serum levels of zinc were low, establishing a diagnosis of AE. The lesions started to subside immediately after administering oral zinc therapy at a 5 mg/kg/day dose. A similar, milder case in an infant presented after weaning with anogenital, perioral dermatitis, and diarrhea. It responded well to 3 mg/kg/day oral zinc therapy. Biotin deficiency is a nutritional dermatosis with varied clinical manifestations. It presents mainly with dermal and neurological features and mimics zinc deficiency. A five-year-old male child presented with eczematous plaques, recurrent infections, numbing and tingling sensations in the lower extremities, and alopecia. Both AE and biotin deficiency were suspected, and on further evaluation, the patient showed normal zinc and biotin levels. Urinary examination revealed increased excretion of 3-hydroxyisovaleric acid in the urine, which is a reliable marker for determining biotin status. Hence, a diagnosis of biotin deficiency was established, and lesions were reduced in a couple of weeks after oral biotin supplements were started [24].

The pediatric population is more susceptible to cutaneous adverse drug reactions (CADR) due to the use of unlicensed and off-label drugs [25]. Few other studies have shown a higher prevalence of CADR in children with polypharmacy, mostly involving psychotropic medications [26]. A case of toxic epidermal necrolysis (TEN) and drug reaction with eosinophilia and systemic symptoms (DRESS) presented to us secondary to phenytoin and amoxicillin antibiotics, respectively. The mortality rates in Stevens-Johnson syndrome (SJS) and TEN cases are higher, and hence, multidisciplinary care in the pediatric ICU to prevent infections and maintain hydration and nutrition is paramount. Shah et al. reported five cases of CADR, including three cases of maculopapular rash, one case of each of erythema multiforme, and SJS. Mathias reported 12 (13%) cases of drug reactions, and nine of them were SJS/TEN [9].

In other or miscellaneous conditions, the majority of cases were IH for systemic propranolol therapy. Approximately 10% of vascular tumors, such as port-wine stain (PWS) and hemangiomas, can cause significant morbidity through uncontrollable bleeding, ulcerations, airway obstruction, ocular compression, and functional impairment [27]. We reported nine cases of hemangioma in infants; four of those presented with a history of recurrent bleeding, and two had ulcerations. Non-selective beta blocker propranolol was commenced on the initial dose of 0.5 mg/kg/day in three divided doses, and the dose was increased to 1 mg/kg/day and eventually to 2 mg/kg/day after three days and seven days, respectively. The patients were carefully monitored for hypotension and hypoglycemia after administration of the drug. All the patients tolerated the dose well without any side effects, and the average duration of hospitalization was 3.5 days.

Primary immunodeficiency diseases (PIDs) are a group of genetic disorders characterized by an increased risk of infections, autoimmunity, malignancy, and skin manifestations [28]. An eight-year-old child presented with recurrent pulmonary infections, coarse facial features, a cold abscess, and eczematous lesions over the extremities. Hyper IgE syndrome was suspected, and genetic testing confirmed the STAT3 mutation in the child. A broad-spectrum antibiotic was started, and baricitinib, a Janus kinase (JAK) inhibitor, was given 2-4 mg/day for four months.

Neonatal lupus erythematosus (NLE) is a spectrum of cutaneous, cardiac, and systemic manifestations seen in a neonate born to a mother with autoantibodies against Ro/Sjögren's-syndrome-related antigen A (SSA) and La/Sjögren's-syndrome-related antigen B (SSB) [29]. A neonate presented with multiple erythematous, annular plaques over the trunk, and the mother had a known case of systemic lupus erythematosus for five years. The serum antibodies against Ro (SSA) and La (SSB) antigens were positive in the child, confirming a diagnosis of NLE.

In our study, along with PDEs, we focused on patients who had extensive, recurrent lesions with poor quality of life. Some cases are difficult to assess on an outpatient basis and need thorough evaluation as an inpatient, as well as monitoring the side effects of the therapy. Few dermatological conditions look benign during initial evaluation but can soon progress to life-threatening acute skin failure within a few days. Hence, pediatricians and emergency physicians should be sensitized to certain dermatological conditions for early diagnosis and management.

Limitation

The sample size of the study was small, and it was a single-center study of short duration. We did not assess the severity of inpatient children using Nelson’s scoring system. Hence, we advise a large study sample with a severity scoring scale to assess children at the time of presentation should be done in future studies.

## Conclusions

Inflammatory conditions like psoriasis were the most common conditions encountered, followed by hereditary conditions such as keratinization disorders in a semi-urban city in north Karnataka. School-going children were the most common age group in which dermatological emergencies were developed. Hereditary skin disorders need a thorough evaluation to establish a diagnosis so that parents can be educated about the nature of the disease and counseled about future pregnancies. PDEs need interdisciplinary care involving dermatologists, pediatricians, and primary care/emergency physicians. Non-dermatologists need to be sensitized to some of the common pediatric skin emergencies to make early diagnoses and treatments. In the future, dermatologists must be proficient in severity scoring scales and intensive care practices to effectively handle pediatric dermatology emergencies.
